# Supplementary figures and images for: Can you make morphometrics work when you know the right answer? Pick and mix approaches for apple identification
Source: PLoS One. 2018 Oct 15;13(10):e0205357. doi: 10.1371/journal.pone.0205357 (PMC6188776; doi:10.1371/journal.pone.0205357)

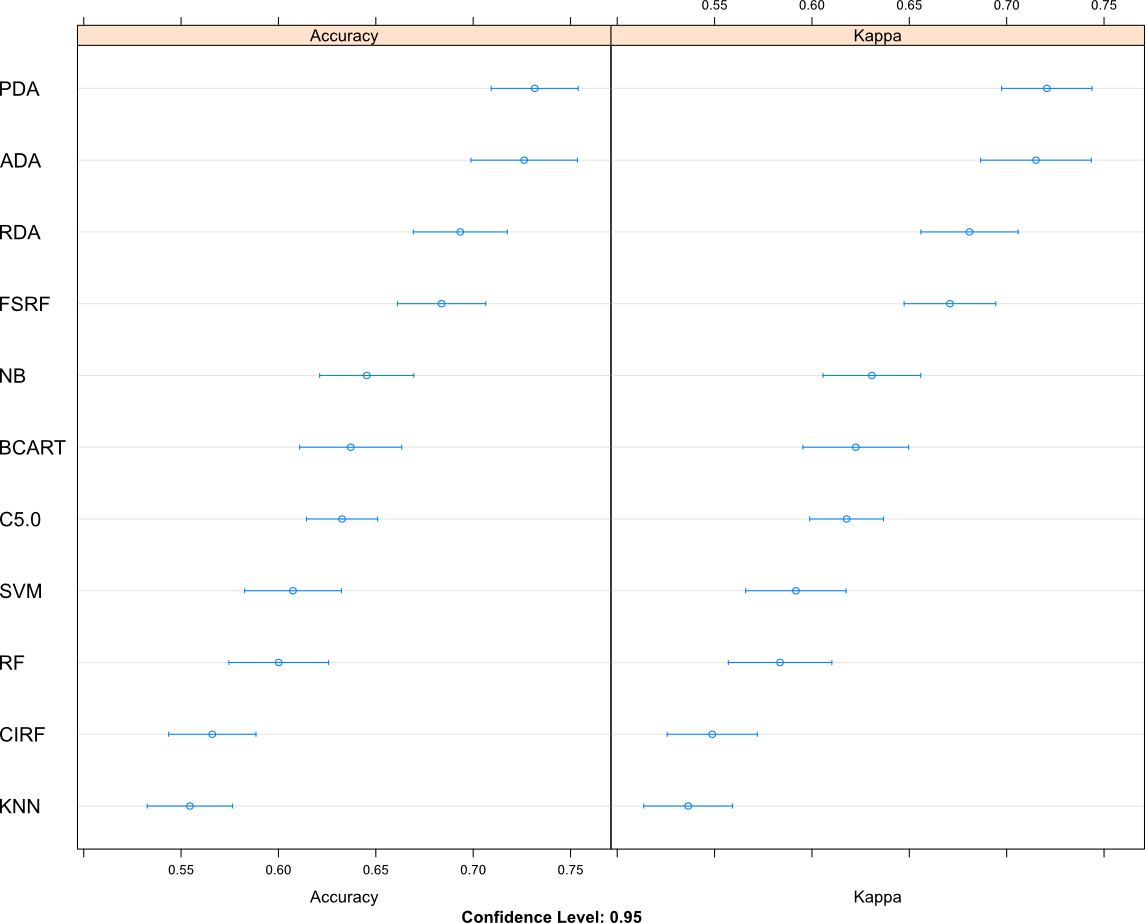

Supplement: S1 Fig — (DOCX) [file pone.0205357.s001.docx]

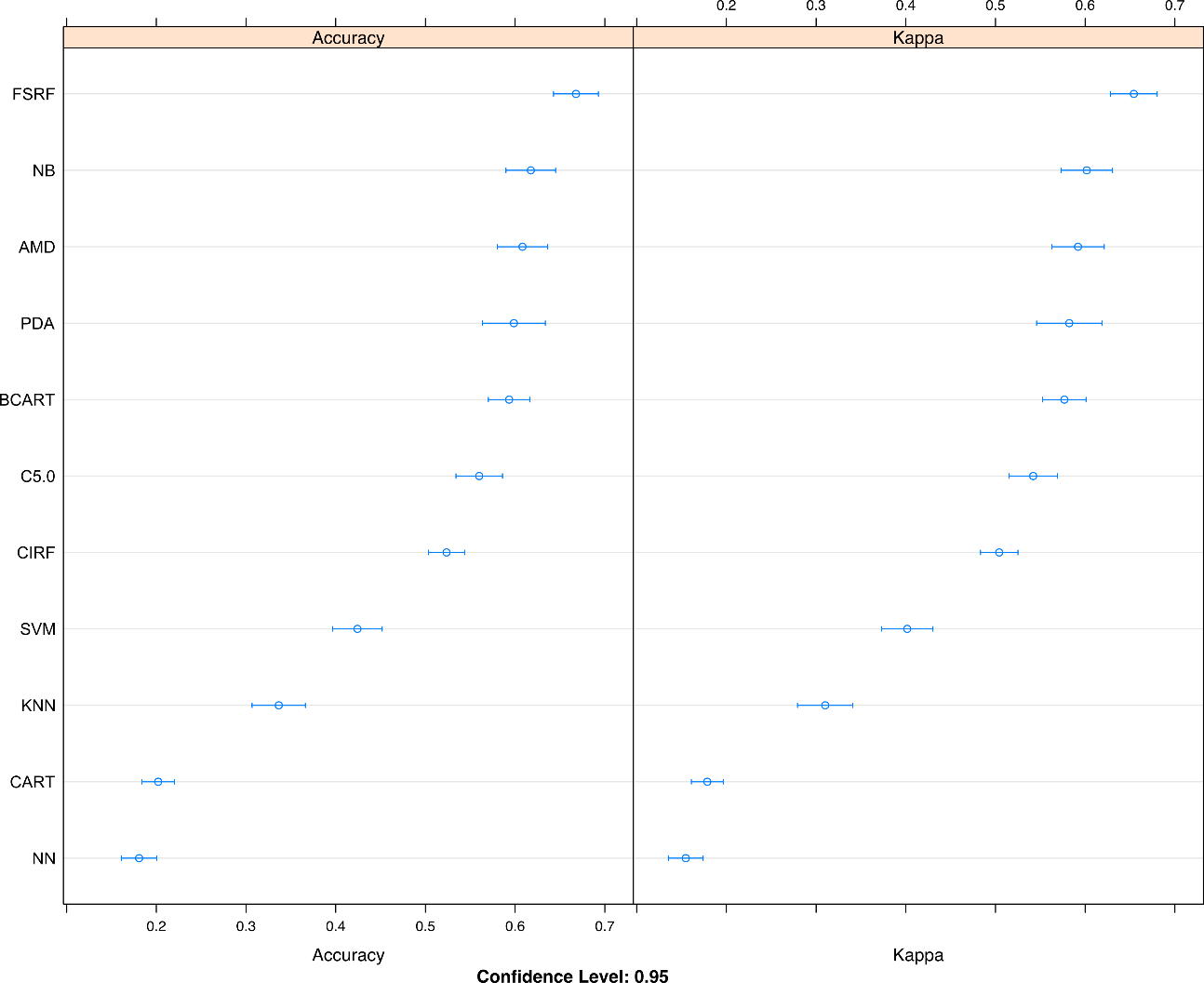

Supplement: S2 Fig — (DOCX) [file pone.0205357.s002.docx]
