# Supplementary material for: Can you make morphometrics work when you know the right answer? Pick and mix approaches for apple identification
Source: PLoS One. 2018 Oct 15;13(10):e0205357. doi: 10.1371/journal.pone.0205357 (PMC6188776; doi:10.1371/journal.pone.0205357)
Supplement: S1 Table — (DOCX) [file pone.0205357.s003.docx]

| Cultivar | Abbreviation | Harvest Date |
| --- | --- | --- |
| ‘Adam’s Pearmain’ | Ada | 23/09/2013 |
| ‘Arlet’ | Arl | 11/09/2014 |
| ‘Beacon’ | Bea | 02/09/2013 |
| ‘Boiken’ | Boi | 07/10/2013 |
| ‘Bovarde’ | Bov | 07/10/2013 |
| ‘Braeburn’ | Bra | 11/09/2014 |
| ‘Burgundy’ | Bur | 11/09/2014 |
| ‘Catshead’ | Cat | 23/09/2013 |
| ‘Charden’ | Cha | 11/09/2014 |
| ‘Cloden’ | Clo | 11/09/2014 |
| ‘Cox’s Orange Pippin’ | Cox | 11/09/2014 |
| ‘Florina’ | Flo | 11/09/2014 |
| ‘Fuji’ | Fuj | 07/10/2013 |
| ‘Golden Delicious’ | Gol | 11/09/2014 |
| ‘Granny Smith’ | Gra | 11/09/2014 |
| ‘Jonathan’ | Jon | 11/09/2014 |
| ‘Kaiser Franz Joseph’ | Kai | 23/09/2013 |
| ‘Liberty’ | Lib | 11/09/2014 |
| ‘Limoncella’ | Lim | 07/10/2013 |
| ‘McIntosh’ | McI | 11/09/2014 |
| ‘Present van Engeland’ | Pre | 23/09/2013 |
| ‘Red Fortune’ (Sport of ‘Fortune’) | Red | 02/09/2013 |
| ‘Rheinischer Krummstiel’ | Rhe | 07/10/2013 |
| ‘Sir Prize’ | Sir | 11/09/2014 |
| ‘Starking’ (Sport of ‘Delicious’) | Sta | 11/09/2014 |
| ‘Vista Bella’ | Vis | 17/07/2014 |
| ‘Wheeler’s Russet' | Whe | 07/10/2013 |
