# Supplementary material for: Can you make morphometrics work when you know the right answer? Pick and mix approaches for apple identification
Source: PLoS One. 2018 Oct 15;13(10):e0205357. doi: 10.1371/journal.pone.0205357 (PMC6188776; doi:10.1371/journal.pone.0205357)
Supplement: S3 Table — The results above the diagonal (represented by grey cells) were derived from t-tests on the accuracy values. The results below the diagonal were derived from t-tests on the kappa values. Significance is indicated with * levels (NS: Not Significant, *:p≤0.05, **≤0.01, ***≤0.001). (DOCX) [file pone.0205357.s005.docx]

|  | | t-tests on accuracy | | | | | | | | | | |
| --- | --- | --- | --- | --- | --- | --- | --- | --- | --- | --- | --- | --- |
|  |  | AMD | PDA | NB | FSRF | CIRF | C5.0 | BCART | KNN | SVM | NN | CART |
| t-tests on kappa | AMD |  | NS | NS | NS | * | NS | NS | *** | *** | *** | *** |
|  | PDA | NS |  | NS | NS | * | NS | NS | *** | *** | *** | *** |
|  | NB | NS | NS |  | NS | *** | NS | NS | *** | *** | *** | *** |
|  | FSRF | NS | NS | NS |  | *** | *** | * | *** | *** | *** | *** |
|  | CIRF | * | * | *** | *** |  | NS | * | *** | *** | *** | *** |
|  | C5.0 | NS | NS | NS | *** | NS |  | NS | *** | *** | *** | *** |
|  | BCART | NS | NS | NS | * | * | NS |  | *** | *** | *** | *** |
|  | KNN | *** | *** | *** | *** | *** | *** | *** |  | *** | *** | *** |
|  | SVM | *** | *** | *** | *** | *** | *** | *** | *** |  | *** | *** |
|  | NN | *** | *** | *** | *** | *** | *** | *** | *** | *** |  | NS |
|  | CART | *** | *** | *** | *** | *** | *** | *** | *** | *** | NS |  |
