# Supplementary material for: Can you make morphometrics work when you know the right answer? Pick and mix approaches for apple identification
Source: PLoS One. 2018 Oct 15;13(10):e0205357. doi: 10.1371/journal.pone.0205357 (PMC6188776; doi:10.1371/journal.pone.0205357)
Supplement: S6 Table — All cultivars with at least a single misclassification are included. The highest three percentages for each cultivar are included. If the correct classification is not in the top three then it is also included together with its rank. If the fruit was correctly classified only the correct classification posterior is included. (DOCX) [file pone.0205357.s008.docx]

| Cultivar | Sample 1 | Sample 2 | Sample 3 | Sample 4 | Sample 5 |
| --- | --- | --- | --- | --- | --- |
| Kai | 1: Red (95.56%)  2: Kai (2.35%) | 1: Red (70.94%)  2: Kai (23.98%) | 1: Kai (77.63%) | 1: Kai (99.44%) | 1: Kai (76.87%) |
| Lib | 1: Sta (34.28%)  2: Lib (26.65%) | 1: Bra (33.2%)  2: Lib (28.24%) | 1: Lib (53.28%) | 1: Lib (51.97%) | 1: Lib (64.80%) |
| Pre | 1: Lim (51.72%)  2: Pre (48.21%) | 1: Pre (99.99%) | 1: Pre (53.98%) | 1: Pre (99.99%) | 1: Pre (99.99%) |
| Sir | 1: Gol (45.35%)  2: Gra (27.82%)  3: Cox (13.58%) | 1: Gol (38.08%)  2: Gra (35.65%)  3: Sir (19.11%) | 1: Sir (96.34%) | 1: Sir (96.25%) | 1: Sir (82.12%) |
|  | 7: Sir (1.09%) |  |  |  |  |
| Sta | 1: Arl (74.73%)  2: Lib (13.47%)  3: Sta (5.62%) | 1: Sta (34.69%) | 1: Sta (53.33%) | 1: Sta (33.02%) | 1: Sta (73.76%) |
| Vis | 1: Arl (35.36%)  2: Sta (15.39%)  3: Bur (14.29%) | 1: Jon (30.2%)  2: Bur (23.4%)  3: Lib (9.88%) | 1: Lib (27.67%)  2: Jon (20.94%)  3: Arl (15.93%) | 1: Vis (83.98%) | 1: Vis (71.37%) |
|  | 9: Vis (1.07%) | 9: Vis (1.82%) | 6: Vis (7.76%) |  |  |
| Whe | 1: Boi (60.71%)  2: Bov (26.43%)  3: Whe (9.08%) | 1: Whe (99.99%) | 1: Whe (99.18%) | 1:Whe (99.92%) | 1:Whe (77.22) |
