# Supplementary material for: Can you make morphometrics work when you know the right answer? Pick and mix approaches for apple identification
Source: PLoS One. 2018 Oct 15;13(10):e0205357. doi: 10.1371/journal.pone.0205357 (PMC6188776; doi:10.1371/journal.pone.0205357)
Supplement: S9 Table — Classifier abbreviations follow Table 1. (DOCX) [file pone.0205357.s011.docx]

| Classifier | Accuracy | Kappa value |
| --- | --- | --- |
| SVM | 27.4% | 0.246 |
| PDA | 26.7% | 0.238 |
| KNN | 25.9% | 0.231 |
| CIRF | 25.2% | 0.223 |
| C5.0 | 21.5% | 0.185 |
| FSRF | 18.5% | 0.154 |
| NN | 7.4% | 0.038 |
